# Risk factors for kidney cancer and socio-occupational category: significant impact of chlorinated solvents (UroCCR 111)

**DOI:** 10.1007/s00345-024-05356-9

**Published:** 2024-11-21

**Authors:** Matthieu Ferragu, Jean-Christophe Bernhard, Alexis Fontenil, Julien Guillotreau, Frédéric Panthier, Nicolas Branger, Olivier Belas, Jean-Jacques Patard, François Audenet, Louis Surlemont, Richard Mallet, Thibaut Waeckel, Pierre Bigot

**Affiliations:** 1 Department of Urology, Angers University Hospital, Angers, France; 2 Department of Urology, Bordeaux University Hospital, Bordeaux, France; 3 Department of Urology, Nîmes University Hospital, Nîmes, France; 4 Department of Urology - Pasteur Clinic Toulouse, Toulouse, France; 5 Department of Urology, Tenon Hospital, Paris, France; 6 Department of Urology - Paoli Calmettes Institute, Marseille, France; 7 Pôle Santé Sud - Private Clinic, Le Mans, France; 8 Department of Urology, Mont-de-Marsan Hospital, Mont-de-Marsan, France; 9 Department of Urology, Hôpital Européen Georges Pompidou, Paris, France; 10 Department of Urology, Rouen University Hospital, Rouen, France; 11 Department of Urology, Francheville Polyclinic, Francheville, France; 12 Department of Urology, Caen Normandy University Hospital, Caen, France; 13 Member of Cancerology Committee of the French Association of Urology (CCAFU), maison de l’urologie, 11, rue Viète, Paris, 75017 France

**Keywords:** Epidemiology, Kidney cancer, Risk factors, Chlorinated solvents, Socio-occupational category

## Abstract

**Introduction:**

The rising incidence of renal cell carcinoma (RCC) is a significant concern in cancer research. This study analyses the characteristics of RCC patients based on their socio-professional category and explores the role of chlorinated solvents as a risk factor.

**Materials and methods:**

A multicentre, descriptive epidemiological study was conducted using the UroCCR database. All patients from participating centres who had been diagnosed with RCC between July 2021 and February 2023, as well as those seen for follow-up consultation during this period, were included. Patients were categorised into 5 socio-professional groups based on INSEE’s Profession and Social Categories classification. The characteristics and risk factors of RCC for each group were compared. Binary logistic regression was used to study the exposure to chlorinated solvents and risk factors for clear cell RCC (ccRCC).

**Results:**

A total of 1252 patients were included. Males made up 69.6% of the population. The median age was 64 years, and 87% of the patients had at least one RCC risk factor. ccRCC, papillary, and chromophobe types accounted for 78%, 14.9%, and 8.5% of the population, respectively. The median tumor size was 4.5 cm (SD = 3.3). Farmers had a higher prevalence of ccRCC (91.3%; *p* = 0.05) and larger tumors (median = 6 cm SD = 3.23; *p* = 0.038) than patients from other populations. Smoking and obesity rates were lower (10.1%; *p* < 0.001; 15.9%, *p* = 0.018, respectively), but exposure to chlorinated solvents was higher (50.7%; *p* < 0.001). Exposure to chlorinated solvents was independently associated with higher TNM stages (*p* = 0.044, OR = 1.41 CI (1.01; 1.96)). Obesity and exposure to chlorinated solvents were independent risk factors for ccRCC (*p* = 0.006, OR = 1.6 CI (1.1;2.2) and *p* = 0.028, OR = 1.6 CI (1.1;2.6), respectively).

**Conclusion:**

This study shows the influence of socio-professional categories on exposure to RCC risk factors and tumor characteristics. In particular, farmers stood out from the rest of the study population. Their significant exposure to chlorinated solvents could be an interesting factor to investigate.

## Patient summary

The report examines the risk factor and characteristics of kidney cancer in a French kidney cancer population. Farmers had larger tumors, a higher proportion of clear cell renal carcinoma (ccRCC), and significant exposure to chlorinated solvents. Exposure to chlorinated solvents could be associated with advanced stages. Obesity and exposure to chlorinated solvents could be independent risk factors for ccRCC.

## Introduction

Renal cell carcinoma (RCC) is the 14th most common cancer globally [1]. The highest incidence rates are found in developed regions such as North America and Europe, among men, and particularly among African Americans [2]. The reasons behind this distribution remain unexplained. Investigating the mechanisms underlying these geographic, ethnic, and gender disparities is a priority for the International Agency for Research on Cancer (IARC). In France, data from the National Cancer Institute (INCa) for 2023 reported 17 141 new cases of RCC. The incidence has steadily increased over the past 30 years. Numerous countries have also observed this upward trend [3]. Analyzing this rising and heterogeneous trend presents a number of challenges and is a topic of debate. Part of this increase may be due to the incidental detection of renal masses during more common abdominal imaging examinations in some countries [4]. However, differences between the incidence rates reported in countries with similar healthcare and screening provisions suggest that over-detection is not the sole factor [5]. A global increase in RCC risk factors such as obesity, hypertension, chronic kidney diseases, and smoking also contributes to the rise in RCC, particularly in developing countries [2, 6, 7]. Other known risk factors such as age, male gender, family history, and exposure to trichloroethylene also need to be considered [2, 8]. The latter, a chlorinated solvent used in industrial degreasing, has been clearly identified as an occupational risk factor for RCC. For instance, the French social security code has recognized RCC as an occupational disease since 2021, attributing it to occupational exposure to trichloroethylene. Researchers are suspecting and studying other risk factors such as exposure to arsenic, cadmium, lead, ionizing radiation, pesticides, and organic and chlorinated solvents [9–14].

RCC incidence is expected to continue rising, highlighting the need to identify risk factors and underlying biological mechanisms for effective prevention and screening. Studies have sought to pinpoint high-risk populations, especially within certain professional categories. For instance, a recent cohort study of 14.9 million individuals in Nordic countries revealed that welders, sailors, and security professionals were high-risk groups [15]. In Central and Eastern Europe, another case-control study identified high-risk populations such as farmers, breeders, architects, and mechanical engineers [16]. These variations in risk populations might be due to different exposures to lifestyle-related risk factors or regulations on chemical products that depend on the country in question.

In France, epidemiological studies of RCC risk factors and the identification of high-risk occupational groups are scarce. We conducted a multicentric epidemiological study in France to explore the risk factors and tumor characteristics of RCC patients based on occupational categories. The primary goal was to identify categories more exposed to risk factors and their association with specific types of RCC. In our study population, we also assessed the impact of chlorinated solvents on tumor features and sought to pinpoint specific risk factors for clear cell RCC (ccRCC).

## Methods

This descriptive and multicentric epidemiological study was conducted within the framework of the UroCCR project (NCT03293563), which is IRB-approved and obtained the CNIL authorization number DR-2013-206. The UroCCR project is a multicentric French database established in 2011 and maintained prospectively for patients treated for RCC. All patients received oral and written information about the objectives and methodology of the UroCCR project and written consent was obtained. This study, identified as ancillary UroCCR project 111, was approved by an internal review committee and adheres to the principles of the Helsinki Declaration.

### Population

All centers participating in the UroCCR project were invited to participate in the study. In total, 13 centers agreed to participate. We included all patients diagnosed with RCC between July 2021 and February 2023, as well as patients attending their follow-up consultation during this period. The only exclusion criterion was the absence of pathological RCC confirmation.

### Variables

Several variables were extracted from the database, including patient characteristics (age at diagnosis, gender), known RCC risk factors (BMI, obesity, former or current smoking, hypertension, CKD defined by a creatinine clearance < 60 ml/min/1.73 m², family history of kidney cancer), and tumor characteristics (tumor size, TNM stage, ISUP grade, histology). We assessed occupational exposure to chlorinated solvents during individual interviews. We provided a list of chlorinated solvents studied in RCC to aid patients, including trichloroethylene, perchloroethylene, methylene chloride, tetrachloromethane, trichloromethane, and organochlorine pesticides.

### Socio-professional categories

We collected details of professional backgrounds during individual interviews. When patients had multiple professions, we recorded the one they had practiced the longest. According to INSEE’s 2003 Profession and Socio-professional Category (PSC) measures, we classified patients into 5 socio-professional categories: (1) without professional activity; (2) farm operators; (3) craftsmen, traders, and business leaders; (4) executives and intermediate professions; (5) employees and workers.

### Statistical analyses

We recorded the extracted data in an Excel file and conducted the analyses using IPSS software (IBM, Armonk, NY, USA). Descriptive statistics, including ANOVA and chi-square tests, were used to present and analyze quantitative and qualitative variables. Binary logistic regression models were used to analyze both tumor characteristics associated with chlorinated solvents and risk factors for ccRCC in the studied population.

## Results

### Descriptive statistics of the study population

We included a total of 1252 patients. Men represented 69.6% of the population. The median age at diagnosis was 64 years (SD 11.8 years). The most common risk factor was hypertension (HTA) with 49.9% of patients, and smoking was in second place (27.9%). This was followed by obesity (26.7%), occupational exposure to chlorinated solvents (13.7%), chronic kidney disease (CKD) (5.8%), and a family history of kidney cancer (3.3%). In all, 87.5% of patients had at least one RCC risk factor, and 20.4% had at least three associated risk factors. The proportions of ccRCC, papillary RCC, and chromophobe RCC were 78%, 14.9%, and 8.5%, respectively. There were also two patients with MITF translocation RCC and one with Bellini RCC. An ISUP score > 2 was observed in 46.2% of the population and a TNM score > T2 in 36.6% of individuals. Only 4% of the population had metastases. The median tumor size was 4.5 cm (SD 3.3 cm).

### Comparison of socio-professional categories

**Fig. 1:**
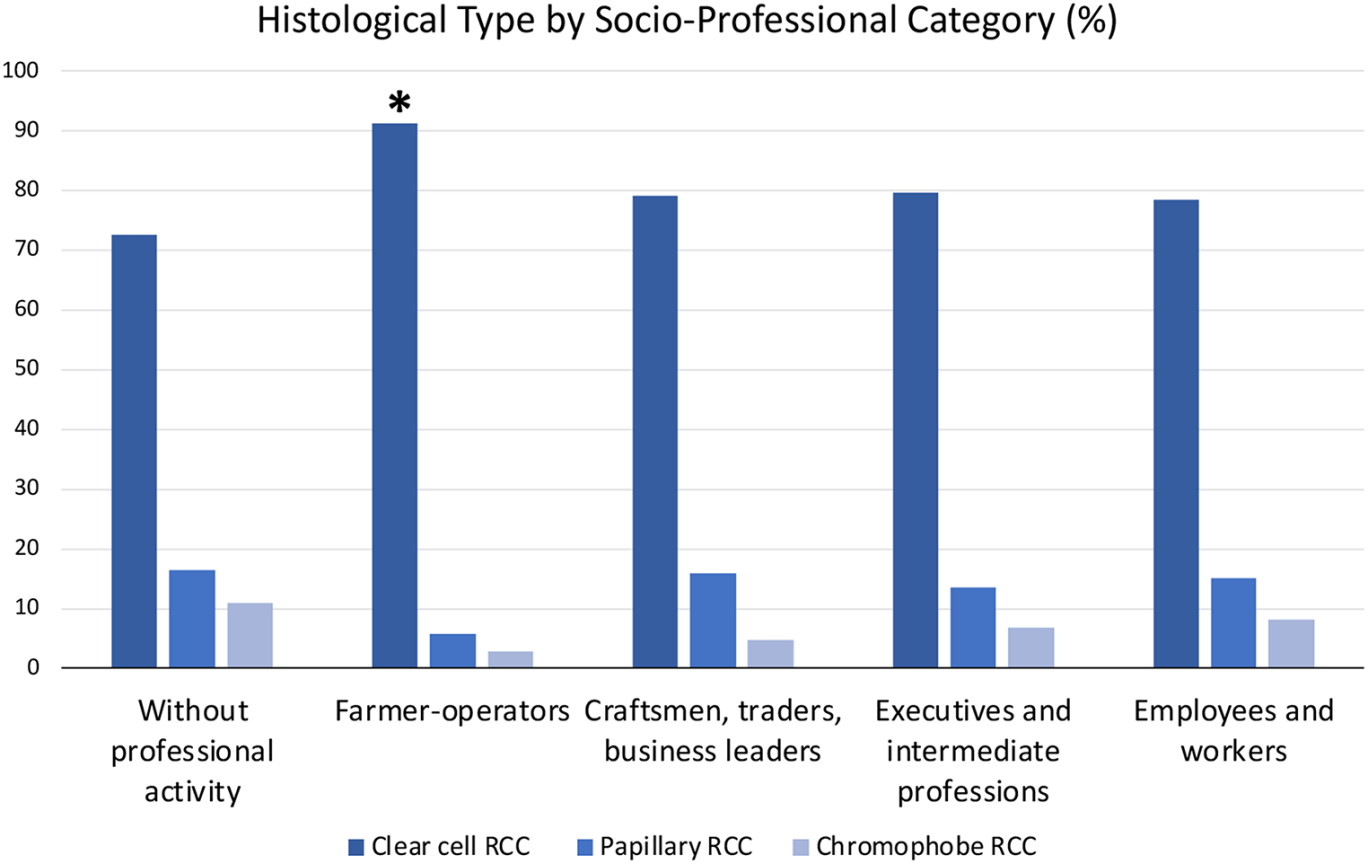
Comparison of histological types by socio-professional category. Farmer-operators had a statistically higher proportion of ccRCC compared to all other categories

**Fig. 2:**
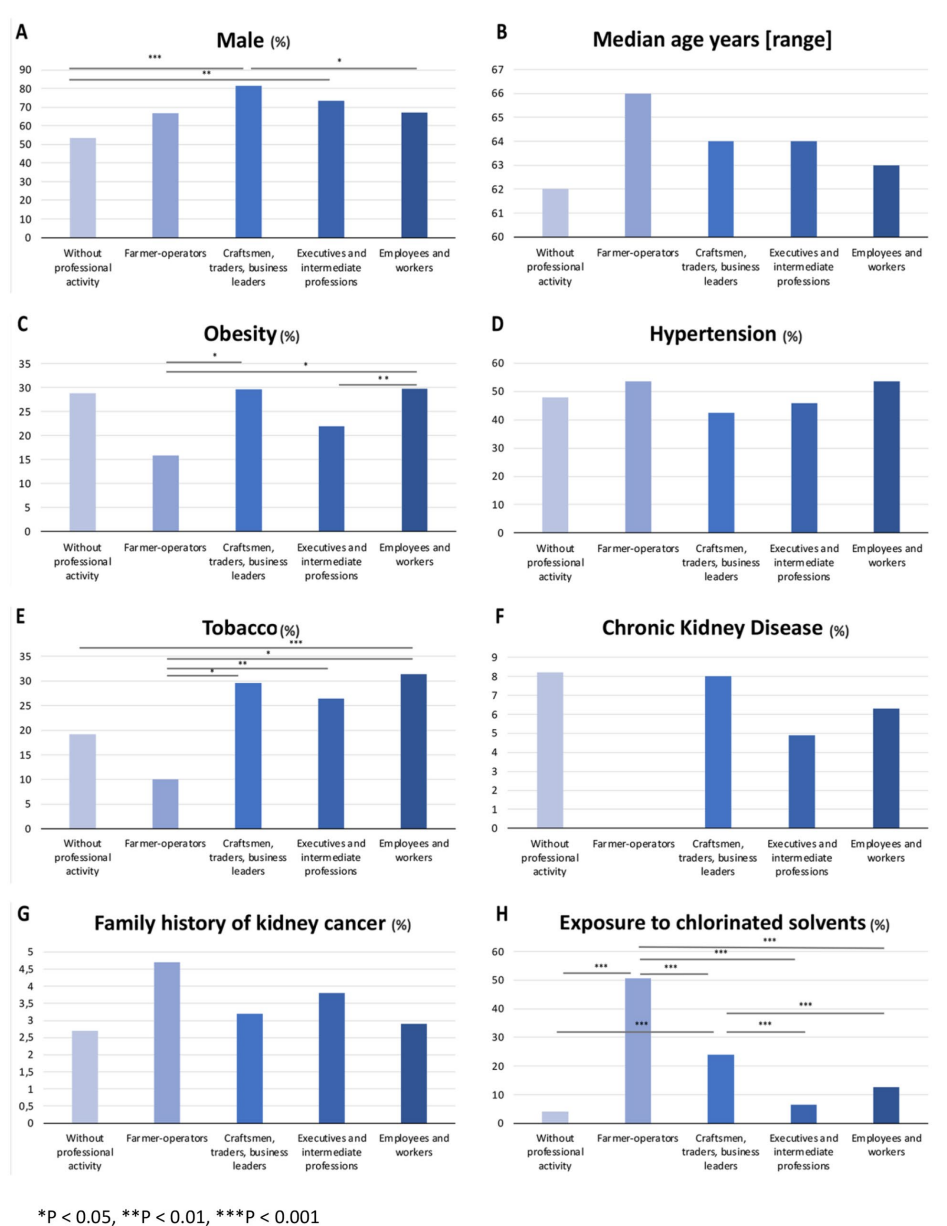
Comparison of RCC risk factors by socio-professional category. (**A**) Male. (**B**) Median age. (**C**) Obesity. (**D**) Hypertension. (**E**) Tobacco. (**F**) Chronic kidney disease. (**G**) Family history of kidney cancer. (**H**) Exposure to chlorinated solvents

In farm operators, tumors tended to be larger (median 6 cm, SD 3.2 cm, *p* = 0.038, Table 1), and the proportion of ccRCC was the highest (91.3%, *p* = 0.05, Table 1; Fig. 1). This group reported the highest exposure to chlorinated solvents (50.7%, *p* < 0.001, Table 1; Fig. 2H), while smoking and obesity were lower (respectively 10.1%, *p* < 0.001, and 15.9%, *p* = 0.018, Table 1; Fig. 2E and C).

**Table 1:**
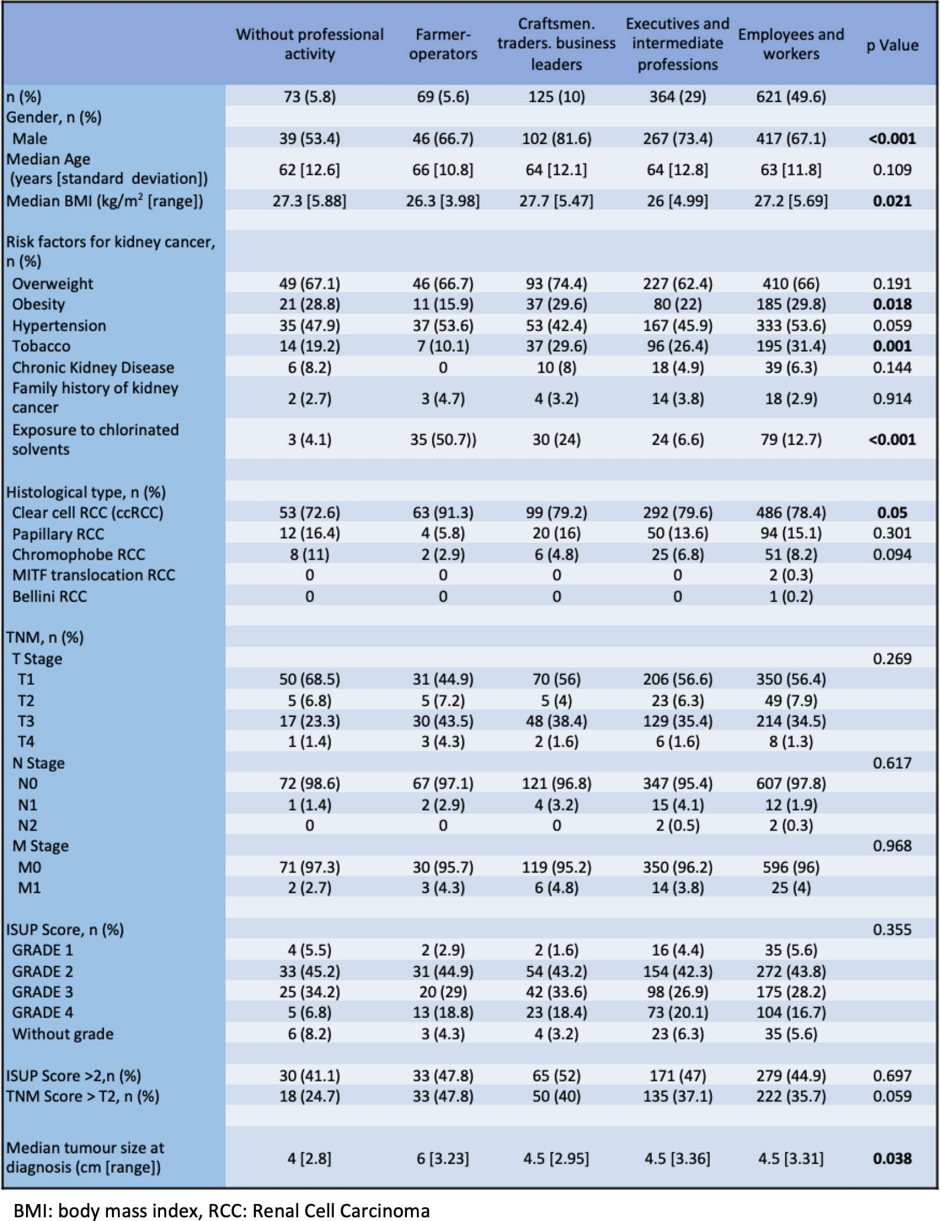
Characteristics of patients according to their socio-professional categories

The group without professional activities had the lowest proportion of men (53.4%, *p* < 0.001, Table 1; Fig. 2A) and smaller tumor sizes (median 4 cm, SD 2.8 cm, *p* = 0.038). The exposure to chlorinated solvents in this category was the lowest (4.1%, *p* = 0.001).

Craftsmen, traders, and business leaders were overwhelmingly men (81.6%, *p* = 0.001). The proportion of smokers, obese patients, and those exposed to chlorinated solvents in this category was significant (respectively 29.6%, *p* = 0.001, 29.6%, *p* = 0.018, and 24%, *p* < 0.001).

Executives and intermediate professions had low exposure to chlorinated solvents (6.6%, *p* < 0.001).

Employees and workers contained the highest proportion of smokers (31.4%, *p* < 0.01), obese patients (29.8%, *p* = 0.018), and hypertensive patients (49.9%, non-significant result, Table 1; Fig. 2D).

There was no statistically significant difference concerning age at diagnosis, TNM stage, and ISUP score (Table 1; Fig. 2B).

### Tumor characteristics associated with exposure to chlorinated solvents

In univariate analysis, exposure to chlorinated solvents was significantly associated with the histological type ccRCC (OR = 1.67 CI (1.07; 2.59), *p* = 0.023) and TNM stage higher than stage T2 (OR = 1.5 (1.09; 2.08), *p* = 0.014). In multivariate analysis, only the TNM stage higher than T2 was still significantly linked to being exposed to chlorinated solvents (OR = 1.41 (1.01; 1.96), *p* = 0.044).

### Risk factors for ccRCC in the study population

In univariate analysis, the risk factors for RCC that were significantly associated with ccRCC were obesity (OR = 1.5 CI (1.1; 2.1), *p* = 0.008) and exposure to chlorinated solvents (OR = 1.7 CI (1.1; 2.6), *p* = 0.023). In multivariate analysis, obesity (OR = 1.6 (1.1; 2.2), *p* = 0.006) and exposure to chlorinated solvents (OR = 1.6 (1.1; 2.6), *p* = 0.028) remained risk factors significantly associated with ccRCC.

## Discussion

In this study, we highlighted an uneven distribution of RCC risk factors and tumor characteristics in the population based on socio-professional categories. Notably among independent farmers, who had a higher prevalence of ccRCC and larger tumors. In this group, the proportion of people exposed to chlorinated solvents was the highest. However, farmers presented lower rates of obesity and exposure to smoking.

Previous studies have highlighted that farmers represent a specific population in terms of cancer incidence. The AGRICOH study is an international cohort of over 200,000 farmers [17]. Farmers have a lower overall cancer incidence compared to the general population. However, certain cancers, such as melanoma, prostate cancer, and myeloma, were overrepresented. In this study, the incidence of RCC was slightly lower than that of the general population, although the difference was not statistically significant (standardized incidence ratio of 0.86, 95% confidence interval = 0.74–1.01). The authors’ hypothesis is that the unique lifestyle of agricultural workers, which differs from that of the general population, could be the cause of this heterogeneous distribution of cancers. Farmers in the AGRICOH study have a lower smoking prevalence and higher levels of occupational physical activity, as well as increased exposure to pesticides, fertilizers, chemicals, exhaust fumes from agricultural machinery, and natural radiation. These results are consistent with the conclusions of our study. Farmers have a lifestyle and exposure to cancer risk factors that differ from the rest of the population.

Regarding the incidence of RCC among farmers, the results of various studies are variable. The aforementioned AGRICOH study observed a slightly reduced risk of RCC. However, other studies conducted in Eastern Europe [16], Montreal [18], and Iowa, USA [19], have reported an increased risk of RCC among farmers. It is interesting to note that the incidence of RCC depends on the geographical area and the population’s risk factors. Therefore, region-specific epidemiological studies are important to examine the risk factors for each population. In our study, we identified a significantly higher prevalence of ccRCC among farmers compared to other socio-professional groups. A case-control study conducted in the United States by S. Karami et al. in 2012 showed similar results [20]. Working in the agricultural sector for 5 years or more was associated with RCC (OR = 3.3 [1.0; 11.5]), particularly with ccRCC (OR = 6.3 [1.7; 23.3]). However, there is a limited number of studies regarding the histological types of RCC in farmers. In France, we can mention the AGRICAN study, which follows more than 180,000 French farmers, but the results regarding the incidence of kidney cancer have not yet been published.

Our study highlights the significant exposure of farmers to chlorinated solvents. These solvents are used as degreasers for agricultural equipment and for the maintenance of agricultural machinery. Their use has decreased since the 1990s, and current trends indicate a likely ban or strict control in the upcoming years. For instance, Europe definitively banned the sale of trichloroethylene in 2016.

One can also highlight the professional exposure of farmers to organochlorine pesticides. These pesticides gained popularity in France starting in the 1940s and were gradually banned between 1980 and 2009. However, a recent French epidemiological study, ESTEBAN, revealed the presence of specific organochlorines formerly used in agriculture in the blood of the current French population [21]. Surprisingly, researchers have detected these chemicals in children’s blood despite their ban for several years. Persistent organic pollutants (POPs) like organochlorine persist in contaminated soils, causing food contamination. The levels of certain organochlorines in the blood samples taken from the farmers were exceptionally high. Prior to the ban, these individuals had direct exposure to these pesticides and are currently operating in areas that remain contaminated. Organochlorine pesticides are suspected to be risk factors for RCC. In a prospective cohort study in 2020, Andreotti et al. analyzed 38 pesticides and their impact on the incidence of RCC in a population of farmers [12]. The results show that 7 of these pesticides were associated with RCC, including 2 organochlorines (2,4,5-T, chlordane) discussed in the aforementioned ESTEBAN study.

Our study indicates an independent association between exposure to chlorinated solvents and ccRCC in a population with RCC. The literature on this subject is quite limited. Few studies examine the risk factors specific to each histological type of RCC. However, exposure to trichloroethylene is associated with a mutation hotspot specific to nucleotide 454 of the VHL gene and is likely linked to ccRCC [22, 23]. Moreover, we found no data in the literature linking exposure to chlorinated solvents with more advanced TNM stage tumors or aggressive criteria for RCC. We need more epidemiological studies to identify the risk factors linked to specific histological types of RCC and their potential influence on cancer aggressiveness.

In terms of the study population, we compared our patients’ socio-professional distribution with the 2022 INSEE data in France. Our study found an overrepresentation of farmers (5.6% compared to 1.6% in the general French population). Our study underrepresented the category of executives and intermediate professions (29% compared to 46.3%). The proportion of workers and employees, as well as that of artisans, merchants, and business owners, were relatively similar. We need additional studies to determine whether these differences stem from changes in RCC risk based on socio-economic status or from sampling fluctuations.

The worldwide increase in the incidence of RCC is a cause for concern [24]. Prevention strategies are being implemented by scientific societies and public authorities to control individual and collective risk factors [25]. Screening for RCC in the general population is another strategy that is attracting growing interest. At the heart of this debate are the issues of cost and selection of patients eligible for screening [26]. Based on this study, we believe that the socio-professional status of patients is a relevant piece of information to be taken into account in preventive actions and in the selection of individuals who would benefit from targeted screening.

This was a descriptive and exploratory study and, as such, has a number of limitations. The retrospective nature of the study introduces recall bias. Patients might, for example, over-report risk factors such as smoking or exposure to chlorinated solvents. Furthermore, we did not know the duration and intensity of the various risk factors. The inclusion period of the study was restricted to almost 2 years (2021–2023), which limited the number of patients. Finally, all patients were diagnosed with RCC, and we did not have a control population to compare our results. That said, this study also has its strengths. The patients came from several types of medical centre (university hospital, non-university public hospital and private clinic) in several French regions. There are few studies in the literature that examine the risk factors for RCC based on socio-professional categories; and, to our knowledge, none has been conducted on a French cohort. It would be relevant to extend the survey to a wider population by assessing the intensity of individual exposure to risk factors for kidney cancer, while exploring in detail the specific effects associated with the various types of chlorinated solvents.

In conclusion, this study demonstrates that socio-professional categories play a significant role in exposure to RCC risk factors as well as in the specific characteristics of tumors. Such variations reflect the distinct lifestyles and occupational exposures of individuals. Our study underscores that exposure to chlorinated solvents has a significant impact on health. Further investigations should be conducted to establish the incidence of RCC according to socio-professional categories, aiming to enhance prevention and specific screening strategies for different populations.

## Data Availability

No datasets were generated or analysed during the current study.
